# Assessing the usefulness of policy brief and policy dialogue as knowledge translation tools towards contextualizing the accountability framework for routine immunization at a subnational level in Nigeria

**DOI:** 10.1186/s12961-021-00804-z

**Published:** 2021-12-30

**Authors:** Lawrence Ulu Ogbonnaya, Ijeoma Nkem Okedo-Alex, Ifeyinwa Chizoba Akamike, Benedict Azuogu, Henry Urochukwu, Ogbonnaya Ogbu, Chigozie Jesse Uneke

**Affiliations:** 1 African Institute for Health Policy and Health Systems, Ebonyi State University, Abakaliki, Nigeria; 2 Department of Community Medicine, Faculty of Clinical Medicine College of Health Sciences, Ebonyi State University, Abakaliki, Nigeria; 3 National Obstetric Fistula Centre (NOFIC), Abakaliki, Nigeria; 4 Department of Applied Microbiology, Faculty of Science, Ebonyi State University, Abakaliki, Nigeria; 5 Department of Medical Microbiology, Faculty of Clinical Medicine, College of Health Sciences, Ebonyi State University, Abakaliki, Nigeria; 6 Department of Community Medicine, Alex Ekwueme Federal University Teaching Hospital, Abakaliki, Nigeria

**Keywords:** Routine immunization, Accountability framework, Policy brief, Policy dialogue, Knowledge translation, Nigeria

## Abstract

**Background:**

Evidence suggests that implementing an accountability mechanism such as the accountability framework for routine immunization in Nigeria (AFRIN) will improve routine immunization (RI) performance. The fact that the AFRIN, which was developed in 2012, still had not been operationalized at the subnational level (Ebonyi State) by 2018 may in part account for the poor RI coverage (33%) in 2017. Knowledge translation (KT) is defined as the methods for closing the gaps from knowledge to practice. Policy briefs (useful in communicating research findings to policy-makers) and policy dialogues (that enable stakeholders to understand research evidence and create context-resonant implementation plans) are two KT tools. This study evaluated their usefulness in enabling policy-makers to contextualize AFRIN in Ebonyi State, Nigeria.

**Methods:**

The study design was cross-sectional descriptive with mixed-methods data collection. A policy brief developed from AFRIN guided deliberations in a 1-day multi-stakeholder policy dialogue by 30 policy actors. The usefulness of the KT tools in contextualizing policy recommendations in the AFRIN was assessed using validated questionnaires developed at McMaster University, Canada.

**Results:**

At the end of the policy dialogue, the policy options in the policy brief were accepted but their implementation strategies were altered to suit the local context. The respondents’ mean ratings (MNR) of the overall usefulness of the policy brief and the policy dialogue in contextualizing the implementation strategies were 6.39 and 6.67, respectively, on a seven-point Likert scale (very useful). The MNR of the different dimensions of the policy brief and policy dialogue ranged from 6.17 to 6.60 and from 6.10 to 6.83, respectively (i.e. moderately helpful to very helpful).

**Conclusion:**

The participants perceived the KT tools (policy brief and policy dialogue) as being very useful in contextualizing policy recommendations in a national policy document into state context-resonant implementable recommendations. We recommend the use of these KT tools in operationalizing AFRIN at the subnational level in Nigeria.

## Background

Routine immunization (RI) remains one of the “best buys” in public health interventions. It is a very cost-effective intervention in preventing child death and eradicating or reducing causes of childhood morbidity [1–3]. According to Liu et al., vaccine-preventable diseases constitute almost one third (29%) of all deaths among under-5-year-old children globally [4]. World Health Organisation (WHO) estimates that immunization prevents 2–3 million deaths every year mainly from diphtheria, pertussis, tuberculosis and measles [5]. Nigeria has the third highest infant and under-5 child mortality rates (70/1000 and 120/1000 live births, respectively) in the world, contributing nearly 10% of the total global burden of child mortality [6]. Research evidence confirms that states and regions of the country with the lowest RI coverage have the highest infant and under-5 mortality rates [7–11]. In Ebonyi State, the infant mortality rate is 67/1000 live births, while the under-5-year mortality rate is 132/1000 live births [11].

Several recent studies have identified lack of accountability and weak governance as an overarching bottleneck contributing to the poor RI performance observed in Nigeria [12–15]. These manifest in ineffective supply chain and logistics, poor service delivery, inadequate human resources, poor data quality, weak demand and funding constraints.

In response to the need for an accountability framework for results-based performance monitoring in RI, the accountability framework for RI in Nigeria (AFRIN) was developed by the Federal Ministry of Health in 2012. This framework was to be adopted and operationalized by the states at the subnational level. The document clearly identified roles and specified who does what in RI management, both across levels of government and within an administrative level. It also specified sanctions and incentives to help enforce the framework. It is envisaged that effective implementation of this framework, especially at the subnational level, will improve RI coverage and lead to better maternal and child health indices in Nigeria. To the best of our knowledge, since the framework was developed, there has been limited operationalization at the subnational level in Nigeria. Anecdotal evidence suggests that the delayed operationalization may have been due to political factors and donor-driven programming of immunization activities.

Knowledge translation (KT) is a process whereby stakeholders are made aware of research evidence and use it to inform decision-making [16]. KT tools such as policy briefs and dialogues have been shown to be useful in facilitating evidence-informed policy development and implementation [17, 18]. Policy briefs are short documents that are used to present the findings and recommendations of a study to readers who area non-specialists [19]. These briefs are recommended mostly as a means of communicating research findings to policy-makers [19]. Policy dialogue in particular is useful in achieving stakeholder understanding of research evidence and drafting of implementation plans. [17].

In initiating the operationalization of AFRIN in Ebonyi State, Nigeria, a policy dialogue structured around a policy brief developed from the AFRIN was convened.

The aim of this study was to evaluate the use of a policy brief and dialogue as KT tools towards contextualizing the AFRIN in Ebonyi.

## Methods

### Description of study area

This study was conducted in Abakaliki, the capital of Ebonyi State, which is one of the five states in the South East geopolitical zone in Nigeria. The state has total land area of 5536 km^2^, with a population in 2017 of 2 943 233, projected from the 2006 census of 2 176 943 at an annual growth rate of 3.2%. With a population density of 390/km^2^, it is one of the most densely populated states in the country. As evidenced by a gross domestic product (GDP) of US $2.732 billion as of 2010 [20], Ebonyi State has made tremendous progress in all aspects of its economy in recent times.

### Study population

The participants included key stakeholders (policy actors) in the RI programme in Ebonyi State. The target group for this study comprised career health policy-makers [21], political (appointed and elected) health policy-makers, state coordinators/heads of United Nations (UN) agencies and other development partners responsible for immunization, health policy and systems researchers from the African Institute for Health Policy & Health Systems, and chief executive officers of member organizations of the Association of Civil Society Organizations for Malaria, Immunization and Nutrition (ACOMIN).

### Study design

This was a cross-sectional descriptive study [22] implemented in two phases:

1. Developing a KT tool (policy brief) from a national policy document—the National Routine Immunization Strategic Plan 2013-2015 (NRISP) containing the AFRIN—and convening a policy dialogue structured on the policy brief to align the policy recommendations in AFRIN to the context of Ebonyi State.
2. Evaluating the usefulness of the KT tools in contextualizing the recommendations in the national policy document.

### Sample size and sampling technique

The study included 30 participants. These were identified and recruited purposively through stakeholder mapping and consultation with key stakeholders in the state RI landscape. Consultations were done using physical visits and phone calls. Although 45 potential participants were identified and invited during the consultation, only 30 participated in the policy dialogue and answered the questionnaire.

#### Development of the policy brief

Possible policy options to enhance accountability in RI implementation as enumerated in the NRISP [23] were identified, and a literature review was carried out to evaluate these options and identify which ones were sufficiently supported by evidence.

The options found to be viable were selected and used to draft the policy brief using the standard techniques for the preparation of policy briefs, meeting the following criteria [19, 24]: provided a persuasive argument, had audience-context specificity, contained actionable recommendations, presented evidence-informed opinions and was written in clear, unambiguous language.

The resulting policy brief, titled “Operationalizing the Accountability Framework for Routine Immunization in Nigeria for Ebonyi State”, recommended eight policy options and implementation strategies (Table 1).

**Table 1:** Policy recommendations in the policy brief titled Operationalizing the Accountability Framework for Routine Immunization in Nigeria for Ebonyi State

No.	Policy options and recommended strategies for implementation
1.	Ensuring that the state cold store, local government area improvised cold stores and RI facilities’ cold chain equipment are adequate and fully functional Strategy: Procurement and installation of solar panels, solar freezers and cold boxes
2.	Ensuring adequate supply of vaccines bundled with auto–disable syringes and needles, and other commodities such as data tools, to all approved RI facilities statewide Strategy: By the push method
3.	Government should motivate skilled health workers, especially in the rural areas Strategy: Through improved regular salaries and payment of rural posting allowances
4.	Government should properly fund RI Strategy: By increasing budgetary allocation and timely release of funds
5.	Government should increase the number of skilled health workers Strategy: Recruit more skilled staff
6.	Timeliness and completeness in data reporting
7.	Strengthening governance for RI accountability
8.	Institutionalization of sanctions and rewards

### Convening the policy dialogue

The policy dialogue was held on 6 March 2018 in a hotel conference hall. The event commenced at 10:30 and ended by 15:30. After registration, the programme for the dialogue event and a fresh copy of the policy brief were distributed to each participant. The policy brief had also been sent to the participants 1 week prior to the session to allow them to familiarize themselves with the policy issue, policy options and the implementation strategies recommended.

The policy dialogue centred on and was guided by the policy brief document. The dialogue process was conducted using the process adopted from Lavis et al. [24], as follows:

(i) Participants were seated at a U-shaped table so each could see the others well and to de-emphasize hierarchy and protocol.
(ii) A welcome address provided the background to the study (lack of accountability in RI programme implementation in the state leading to poor coverage) and the purpose of the policy dialogue (which was to critique and align the recommended policy options and implementation strategies within the context of the state using the participants’ wealth of knowledge in policy-making and tacit knowledge of the state’s cultural and contextual milieu).
(iii) Self-introduction of participants indicating name, organization represented and position in the organization.
(iv) Subdivision of the participants into four groups (ensuring a good mixture of policy-maker, policy implementer, policy researcher, civil society organization activist and development partner official in each group) and assignment of two policy options and their implementation strategies to each group for dialogue in order to identify the best context-sensitive recommendation. The groups reserved the right to adopt all, some or none of the policy recommendations, and were at liberty to appropriately modify the implementation strategies in order to make them “context-sensitive”.
(v) A facilitator with dialogue skills from the African Institute of Health Policy & Health Systems was assigned to each group to moderate the discussion. Each policy option and the implementation strategies were exhaustively deliberated upon.

The small group dialogue sessions lasted for about 2 hours. At the end, participants reconvened in plenary where each group presented their recommendations using flip charts. These were further deliberated upon by the whole house and input received from all other participants. After the presentations, the flip charts were collected from the presenters.

To enhance participants’ capacity to effectively engage in the policy dialogue, a capacity-enhancing session with the theme “the role of policy dialogue in evidence-informed policy-making for RI” was conducted for the 30 participants prior to the commencement of the policy dialogue. This lasted for about 1 hour. There is evidence that skill in policy dialogue participation is enhanced through such capacity-enhancing exercises [25–27].

### Outcome of the policy dialogue

The participants in the policy dialogue adopted all eight policy options recommended in the policy brief and modified the implementation strategies through insightful inputs received during the dialogue. These recommendations, which made the resulting implementation strategies more contextually resonant, are shown in Table 2. For example, participants accepted that government should increase funding for RI but suggested that the Federal Ministry of Health should produce a well-costed annual plan as a basis for evidence-based budgeting, since an evidence-based budget request can hardly be turned down. Participants also adopted the recommendation that government should recruit more skilled staff, but in the interim, human resources for health in the state could be shored up by (i) encouraging volunteerism through mobilizing, incentivizing, training and using educated retired civil servants in their localities for RI services delivery, (ii) refraining from frequent transfers of skilled health workers away from the rural areas, and (iii) providing non-monetary incentives to health workers for work well done, especially those serving in the rural areas. The groups also accepted the need to clearly define and enforce integrated and complementary roles and responsibilities for all stakeholders in the RI sector, including the government, and apply sanctions where any partner failed to perform the assigned roles. They recommended transforming the multi-stakeholder group that participated in the policy dialogue into an accountability “watchdog” group, as only such a group could objectively apply such sanctions without witch-hunting of civil servants (Table 2).

**Table 2:** Comparison of policy options and implementation strategies in the policy brief versus the policy dialogue

No.	Recommended policy options and implementation strategies in the policy brief	Contextualized implementation strategies from the policy dialogue
1.	Ensuring that the state cold store, the local government area (LGA) improvised cold stores and RI facilities’ cold chain equipment were adequate and fully functional Strategy: Procurement and installation of solar panels, solar freezers and cold boxes	Pending the procurement and installation of more solar panels and deep freezers in LGAs and ward health centres, healthcare workers in the LGAs should optimize the use of available solar freezers in baking ice packs by removing and storing baked ice packs in cold boxes, thereby freeing space in the solar freezers for baking new ice packs
2.	Ensuring adequate supply of vaccines bundled with auto–disable syringes and needles, and other commodities such as data tools, to all approved RI facilities statewide Strategy: By the push method	LGAs to submit RI forecast data per antigen and accompanying protocols in a timely manner to the state headquarters to enable proper supply chain planning
3.	Government should motivate skilled health workers, especially in the rural areas Strategy: Through improved regular salaries and payment of rural posting allowances	Governments and communities should motivate health workers who have served well in the rural areas, especially through nonmonetary incentives. for example, communities could confer chieftaincy titles on health workers who have done well in their domain, government could nominate them for state and national awards, and commendation letters could be sent for such workers by the government and communities
4.	Government should fund RI properly Strategy: Through an increase in budgetary allocation and timely release of funds	The Federal Ministry of Health should produce a well-costed annual plan as a basis for evidence-based budgeting, since an evidence-based budget request can hardly be turned down
5.	Government should increase the number of skilled health workers Strategy: Recruit more skilled staff	In the interim, human resources for health in the state could be shored up by (i) encouraging volunteerism by mobilizing, incentivizing, training and using educated retired civil servants in their localities for RI services delivery, and (ii) by refraining from frequent transfers of skilled health workers away from the rural areas
6.	Timeliness and completeness in data reporting	Lack of power for charging phones and laptops and poor road networks hamper data reporting. Intersectoral collaboration between the ministries of health, power, works and information would positively affect RI service delivery. For instance, the Federal Ministry of Power could take over the supply and maintenance of solar panels and solar freezers to the LGA and ward health centres, and priority for road construction could be based on linking ward health centres to the LGA headquarters and state capital
7.	Strengthening governance for RI accountability	The stakeholder group involved in this policy dialogue should not disband but should transform into an independent accountability “watchdog” group that would advocate and ensure accountability in RI programmes
8.	Institutionalization of sanctions and rewards	The government, development partners, healthcare workers, civil society organizations and community members should have defined roles and responsibilities that will be complementary and integrated, and the discharge of such roles and responsibilities should be enforced

### Evaluation of the usefulness of the policy brief and policy dialogue

The policy brief and the policy dialogue process were evaluated using the process adopted from Lavis et al. [24].

The policy brief assessment was conducted before the commencement of the policy dialogue. On arrival, the participants were given 45 minutes to read and acquaint themselves with the discussion points and the objectives of the dialogue, after which they were provided a copy of the policy brief assessment questionnaire to assess the quality of the policy brief document and the relevance of the policy options and implementation strategies recommended for addressing the policy issue at stake.

Similarly, at the end of the policy dialogue, they were provided with the policy dialogue assessment questionnaire to assess the usefulness of the policy dialogue in making the policy recommendations and the implementation strategies in the policy brief context-resonant and therefore implementable.

### Data collection

Data were collected using the policy brief and policy dialogue assessment questionnaires developed by Johnson and Lavis [28]. The quantitative data consisted of response items to the seven-point Likert scale questions. The qualitative data consisted of responses to the open-ended questions in the questionnaire. The questionnaire was divided into three sections, A–C. Section A elicited information on the participants’ profiles including gender, age category, marital status, official designation attributes, educational qualification and level of operation. Section B elicited information on participants’ perceptions of the usefulness of the policy brief and the policy dialogue process. Section C contained the open-ended questions that elicited qualitative data regarding the participants’ views on what could be done better or differently next time and one important action that could be done better or differently to address future policy briefs on the same policy issue.

### Data analysis

The data were analysed using the methods developed at McMaster University, Canada, by Johnson and Lavis [28], based on mean rating (MNR), median rating (MDR) and range (R). The response items in the questionnaire represent a Likert rating scale of 1–7 points, where 1 = very unhelpful, 2 = unhelpful, 3 = slightly unhelpful, 4 = neutral, 5 = slightly helpful, 6  = helpful and 7 = very helpful. The MNR, MDR and R values were rated as follows: 1.00–3.99 was considered low, 4.00–4.49 was considered the natural neutral point and 4.50–7.00 was considered high. The Statistical Package for the Social Sciences (SPSS) version 22 software was used for the data analysis.

## Results

Forty-five policy-makers were mapped for this study, but only 30 (66.67%) attended and participated in the event.

### Profile of participants at the policy dialogue

The results show that a majority (56.7%) of the participants were male, slightly more than half (53.3%) were aged ≥ 45 years and a majority (86.6%) had at least a bachelor’s degree.

The institutions that participated in the policy dialogue included the Ebonyi State Primary Health Care Development Agency, State Ministry of Finance and Economic Development, Ebonyi State House of Assembly–House Committee on Health, development partners, civil society organizations, media organizations and the African Institute for Health Policy & Health Systems Studies. The official designations of the respondents included executive secretaries, directors and heads of units in immunization-related parastatals, legislators, zonal and state team leads of development partner agencies, media representatives and health policy and systems researchers. The mean duration in designated position was 4.98 ± 4.10 years, and 30.0% had a direct influence on policy-making (Table 3).

**Table 3:** Profile of participants at the policy dialogue

Variable	Freq (%) *n* = 30
*Gender*	
Male	17 (56.7)
Female	13 (43.3)
*Age category*	
25–34 years	4 (13.3)
35–44 years	10 (33.3)
≥ 45 years	16 (53.3)
*Marital status*	
Single	2 (6.7)
Married	28 (93.3)
*Highest educational qualification*	
Senior secondary certificate of education (SSCE)/diploma	4 (13.3)
Bachelor’s	12 (40)
Master’s	10 (33.3)
PhD	4 (13.3)
*Institutional affiliation*	
Ebonyi State Primary Health Care Development Agency	9 (30.0)
State Ministry of Finance and Economic Development	5 (16.7)
Ebonyi State House of Assembly–House Committee on Health	3 (10.0)
Development partners	3 (10.0)
Civil society organizations	4 (13.3)
Media organizations	2 (6.7)
African Institute for Health Policy & Health Systems Studies	4(13.3)
*Duration in position (frequency)*	
< 1 year	1 (3.3)
1–5 years	19 (63.3)
6–10 years	5 (16.7)
≥ 10 years	4 (13.3)
No answer	1 (3.3)
*Level of operation (frequency)*	
Primary	12 (40.0)
Secondary	2 (6.7)
Tertiary	8 (26.7)
No answer	8 (26.7)
*Influence in policy-making (frequency)*	
Direct	9 (30.0)
Indirect	17 (56.7)
No answer	4 (13.3)

#### Perception of the respondents on the usefulness of the policy brief

The most highly rated aspects of the policy brief by the respondents were the proper description of the context of the issue being addressed (MNR = 6.60) and considerations for quality when discussing the research evidence (MNR = 6.50). The lowest rated were the policy brief not concluding with particular recommendations (MNR = 5.43) and the policy brief including a graded-entry format (5.89) (Table 4).

**Table 4:** Participants’ view on policy brief design and production

No.	View about how the policy brief was designed and produced	MNR	MDR	Min–Max
1.	The policy brief described the context of the issue being addressed	6.60	7.00	5.00–7.00
2.	The policy brief described different features of the problem, including (where possible) how it affects particular groups	6.43	6.50	5.00–7.00
3.	The policy brief described at least three options for addressing the problem	6.23	6.00	4.00–7.00
4.	The policy brief described what is known, based on synthesized research evidence, about each of the options and where there are gaps in what is known	6.17	6.00	5.00–7.00
5.	The policy brief described key implementation considerations	6.37	6.00	6.00–7.00
6.	The policy brief employed systematic and transparent methods to identify, select and assess synthesized research evidence	6.37	7.00	4.00–7.00
7.	The policy brief took quality considerations into account when discussing the research evidence	6.50	7.00	5.00–7.00
8.	The policy brief took local applicability considerations into account when discussing the research evidence	6.47	7.00	5.00–7.00
9.	The policy brief took equity considerations into account when discussing the research evidence	6.10	6.00	4.00–7.00
10.	The policy brief did not conclude with particular recommendations	5.43	6.00	1.00–7.00
11.	The policy brief employed a graded-entry format (e.g. a list of key messages and a full report)	5.89	6.00	3.00–7.00
12.	The policy brief included a reference list for those who wanted to read more about a particular systematic review or research study	5.87	6.00	4.00–7.00
13.	The policy brief was subjected to a review by at least one policy-maker, at least one stakeholder and at least one researcher (called a “merit” review process to distinguish it from “peer” review, which would typically only involve researchers in the review	6.23	6.00	4.00–7.00
14.	The purpose of the policy brief was to present the available research evidence on a high-priority policy issue in order to inform a policy dialogue where research evidence would be just one input to the discussion	6.39	7.00	4.00–7.00

#### Perceptions of the respondents on the usefulness of the policy dialogue in addressing issues highlighted in the policy brief

The policy dialogue was considered a highly useful approach for discussing a high-priority issue (MNR 6.80) and generating at least three options for solving the problem. All other parameters assessed had MNR of > 6 (Table 5).

**Table 5:** Usefulness of the policy dialogue in addressing issues highlighted in the policy brief

No.	Parameters assessed	MNR	MDR	R
1.	The policy dialogue discussed a high-priority policy issue. How helpful did you find this approach?	6.80	7.00	5–7
2.	The policy dialogue provided an opportunity to discuss different features of the problem, including how it affects infants and under-5-year age groups. How helpful did you find this approach?	6.83	7.00	5–7
3.	The policy dialogue provided an opportunity to discuss at least three options for addressing the problem. How helpful did you find this approach?	6.77	7.00	6–7
4.	The policy dialogue provided an opportunity to discuss key implementation considerations. How helpful did you find this approach?	6.73	7.00	5–7
5.	The policy dialogue provided an opportunity to discuss who might do what differently. How helpful did you find this approach?	6.63	7.00	5–7
6.	The policy dialogue was informed by a pre-circulated policy brief. How helpful did you find this approach?	6.73	7.00	5–7
7.	The policy dialogue was informed by discussion about the full range of factors that can inform how to approach a problem, possible options for addressing it and key implementation considerations. How helpful did you find this approach?	6.70	7.00	5–7
8.	The policy dialogue brought together many parties who could be involved in or affected by future decisions related to the issue. How helpful did you find this?	6.73	7.00	5–7
9.	The policy dialogue aimed for fair representation among policy-makers, stakeholders and researchers. How helpful did you find this approach?	6.70	7.00	5–7
10.	The policy dialogue engaged a facilitator to assist with the deliberations. How helpful did you find this approach?	6.66	7.00	5–7
11.	The policy dialogue allowed for frank, off-the-record deliberations by following the Chatham House rule: “Participants are free to use the information received during the meeting, but neither the identity nor the affiliation of the speaker(s), nor that of any other participant may be revealed. How helpful did you find this approach?	6.60	7.00	5–7
12.	The policy dialogue did not aim for consensus. How helpful did you find this approach?	6.10	7.00	3–7
13.	The purpose of the policy dialogue was to support a full discussion of relevant considerations (including research evidence) about a high-priority policy issue in order to inform action. How well did the policy dialogue achieve this purpose?	6.67	7.00	5–7

#### Participants’ views on how to improve future policy briefs

(i) What needs to be retained: The two commonest features participants wanted retained in future policy briefs were taking cognizance of the local context by 26.32% and the systematic transparent method employed in identifying, selecting and assessing synthesized research evidence by 21% of the respondents. Other features they wished to retain are shown in Table 6.
(ii) What should be changed: The majority (88.9%) suggested nothing to be changed, while 5.6% wanted the policy brief to be shorter to make for easier reading and wanted more local literature incorporated into the evidence synthesis.
(iii) One important action that could be done better or differently to address the featured policy issue: Of the responses to this question, 25% said they would circulate the policy brief much earlier, get input from local researchers in developing the policy brief, make specific recommendations in the policy brief and ensure that more political policy-makers participated in the “merit” review.

**Table 6:** Participants’ views on how to improve future policy brief development and policy dialogue on the same policy issue in the future

Policy brief *n* = 19	Freq (%)	Policy dialogue *n* = 20	Freq (%)
*1. What should be retained*			
Taking cognizance of the local context	5 (26.3)	Participatory and interactive engagement which gave room for each participant to make useful contributions	5 (25)
Employment of systematic transparent method in the identification, selection and assessment of synthesized research evidence	4 (21.1)	Fair representation of stakeholders in the various subgroups during the deliberations	4 (20)
The “merit” review process	2 (10.5)	Involvement of all stakeholders	3 (15)
Description of the current situation in the featured policy issue	2 (10.5)	Well-itemized problems and policy recommendations	2 (10)
Outlined implementation strategies	2 (10.5)	Flexibility in dialogue which led to respect of opinions	2 (10)
Description of the policy document from where the policy brief was developed (AFRIN)	2 (10.5)	Systematic and transparent method that is research evidence-based	2 (10)
Description of the issue to be addressed	1 (5.3)	Frank off-the-record deliberations	2 (10)
Inclusion of references	1 (5.3)		

Policy brief *n* = 18		Policy dialogue *n* = 11	
*2. What should be changed?*			
No changes suggested	16 (88.9)	Nothing	4 (36.4)
Should be shorter with fewer pages to make it easier to read	1 (5.6)	Participation by the apex policy-makers in the ministry in policy dialogues should be mandatory	3 (27.3)
More local literature should be highlighted	1 (5.6)	When subgroups for discussions are created, members should not be randomly allocated	1 (9)
		Adding more details to the description of the context of the problem	1 (9)
		Participants should be informed earlier about the policy dialogue	1 (9)
		The language of discussion should be kept simple for easy understanding	1 (9)

Policy brief *n* = 4		Policy dialogue *n* = 8	
*3. One important action that you personally can do better or differently to address the featured policy issue*			
Circulate the policy brief much earlier	1 (25.0)	Collect, collate and share complete and relevant data in a timely manner	3 (37.5)
Future policy briefs should make specific recommendations	1 (25.0)	Take ownership of the work one is doing, e.g. through personal commitment, to ensure that recommended strategies are implemented	2 (25)
Getting input from local researchers and consider local applicability of their results	1 (25.0)	Cost-effective methods of maintaining cold chain, e.g. by harnessing community resources	2 (25)
Increase the number of political policy-makers in the “merit” review process to at least three	1 (25.0)	Facilitating the “push” programme of vaccine and commodity distribution from the state straight to health facilities	1 (12.5)

#### Participants’ views on how to improve future policy dialogues

(i) What to retain: The features participants most commonly wanted retained in future policy dialogues included the participatory and interactive engagement which gave room for each participant to make useful contributions (25%), the involvement of all stakeholders in the policy dialogue (20%) and the fair representation of stakeholders in the various subgroups during the deliberations (15%).
(ii) What should be changed: Although the largest share (36.4%) were satisfied with the way and manner the policy dialogue was conducted and suggested no changes, 27.3% wanted participation by the political policy-makers made mandatory.
(iii) Important actions that participants were personally willing to do better or differently: Of the eight persons who responded to this question, the largest share (37.5%) committed to collating and disseminating relevant and complete RI data in a timely manner, 25% committed to taking ownership of their work and implementing cost-effective methods of cold chain maintenance, and 12.5% committed to facilitating the “push” programme of vaccine and commodity distribution from the state straight to health facilities (Table 6).

## Discussion

This study evaluated the usefulness of KT tools (policy brief and policy dialogue) in enabling policy-makers in Ebonyi State to contextualize and operationalize a national policy document (AFRIN).

The approach used was informed from the 2018 report of the Global HIV/AIDS Initiatives Nigeria, which recommended that policy briefs be included as part of any comprehensive communication strategy and used for engaging both policy-makers and researchers at the subnational and national levels towards contextualization and operationalization. A key reflection on the role of the policy dialogue in contextualizing the policy options created by the policy brief is seen in the outcome of the policy dialogue, where the stakeholders recommended volunteerism instead of recruitment of skilled workers, nonmonetary compensation of health workers serving in rural communities (such as through conferment of state honours and chieftaincy titles by communities they have served well), optimal use of available solar freezers and other cold chain equipment instead of buying and installing new equipment, and refraining from frequent transfer of skilled health workers away from rural areas by making it statutory that every such worker would have served for at least 3 years before such transfer. These decisions were taken bearing in mind certain realities of the state. First was the financial constraints the state faces as one of the states receiving the smallest allocations from the monthly Federal Account Allocation Committee (FAAC) meeting. This committee decides the amount of funds that each of the 36 states of Nigeria receives from the federal account based on certain fixed criteria. This is compounded by the fact that the state has low internally generated revenue as a result of a weak tax base due to the near absence of industry and other commercial ventures. Second, the health sector both nationally and in the state faces chronic under-funding. The state has never achieved the recommendation of the Abuja Declaration in 2001 by African heads of government that every country should allocate a minimum of 15% of their annual budget to the health sector [29]. Finally, there has been no recruitment of staff into the health sector for about 10 years despite the fact that workers have been retiring from service and dying, so there is an acute shortage of skilled manpower sector-wide.

The use of the policy brief in this study allowed career policy-makers, researchers and other stakeholders in the RI programme to identify the most feasible policy options of the AFRIN for implementation in Ebonyi State. Studies have demonstrated the usefulness of policy briefs and policy dialogue in policy-maker and researcher engagement [26].

Our study is similar to that of Yehia et al. [30], which evaluated the usefulness of KT tools (policy brief and policy dialogue) in the development of mental health policy in Lebanon. In that study, a policy brief developed from a comprehensive evidence synthesis and findings from key informant interviews of 10 policy-makers and key informants defined the problem and suggested three policy options as solutions. This policy brief guided a policy dialogue on mental health services development in Lebanon. Evaluation of the policy dialogue showed that integrating mental health services into primary healthcare was the option most frequently suggested by the 24 participants, thus validating the evidence in the policy brief. A post-dialogue survey conducted 6 months later showed that stakeholders had undertaken many implementation steps including the establishment of a national task force on mental health, training primary healthcare staff on mental health services and updating the national essential drug list to include psychiatric medicines. They concluded that the use of KT tools to help generate evidence-informed programmes was promising in Lebanon.

Johnson et al. [31] reported on the lessons learned from an initiative called Nigeria Research Days (NRD), using policy dialogue in supporting the use of evidence for establishing maternal, newborn and child health policy. The paper described the conceptualization, implementation and conduct of the first edition of the NRD policy dialogue framework. The framework for KT in that study was “the knowledge value chain” framework, a nonlinear concept based on the management of five dyadic capabilities: knowledge mapping and acquisition, knowledge creation and destruction, knowledge integration and sharing/transfer, knowledge replication and protection, and knowledge performance and innovation. The Department of Family Health of the Nigerian Federal Ministry of Health initiated and organized the NRD to serve as a platform for exchange between researchers and policy-makers for improving maternal, newborn and child health. In the first edition of the NRD, a cross-sectional study was designed to assess the effectiveness of a policy dialogue during the NRD. A descriptive analysis of the data collected from the workshop evaluation survey showed that the participants rated the content and format of the meeting positively and made suggestions for improvement. They were willing to implement the recommendations of the final communiqué and concluded that the lessons learned from this first edition would be used to improve future editions. However, unlike in our study, that study did not employ the use of a policy brief; rather, researchers who were implementing grants from the Canadian initiative Innovating for Mother and Child Health in Africa (IMCHA) were mapped and invited to present the outcomes of their studies to the audience of researchers, policy-makers and non-state actors involved in maternal and child health.

Unlike a previous policy dialogue on the control of infectious diseases of poverty in Ebonyi State [26], which involved participation from two institutions, the present study had an interesting feature of having participation from across many sectors. All of the participants were mid-level to senior officers in their respective organizations (career policy-makers), with 86.7% directly or indirectly influencing the policy-making process. As envisaged by Bammers et al. [21], deliberate targeting of such career policy-makers through the kind of enterprise employed in this study will help in bridging the “know–do gap”, as many career civil servants neither recognize nor accept their roles in policy-making. This is despite the high level of influence on policy-making reported by these participants. Anecdotal evidence suggests that career policy-makers, in contrast to their elected and appointed counterparts, tend to be well versed and familiar with routine decision-making processes given their length of time in service, experience, cross-sectoral exposure and continuity in office across various political regimes. This highlights the need for more focus on engaging these career policy-makers on policy-related issues. It is anticipated that with the additional skill enhancement through the workshop on policy dialogue they received, they will realize that they are policy-makers and thereafter play a greater role, both directly and indirectly, in policy-making.

The quality and relevance of the policy brief developed for this study was rated very highly (moderately useful to very useful), which is similar to the finding reported by Uneke et al. [25]. This is probably because similar methods for policy brief preparation as recommended by Lavis et al. and Jones and Walsh [19, 24] were utilized in both studies. Likewise, the participants considered the policy dialogue process and brief to be very useful. Again, this can be explained by the fact that well-established guidelines [24, 32, 33] for the conduct of policy dialogues were followed.

This research compared the policy options in AFRIN as stated in the policy brief and the context-driven suggestions provided by the participants. The key implementation strategies listed centred on supply and logistics, proper budgeting and funding, human resource management, and intersectoral/organizational collaboration and creation of accountability interest/advocacy groups. Proper budgeting and availability of funds has a role in effective implementation of policies/programmes. Regarding human resource management, the gap can be bridged by considering task-shifting, which is less expensive and can contribute to better implementation. Studies have shown the value of task-shifting and equitable urban–rural distribution of health workers in various aspects of health programmes [34].

Policy dialogues provide the much-needed opportunity for policy-makers to review suggested policy directions and proffer revisions and implementation considerations relevant to the particular context. The contextualization of policy options stated in the policy brief during the policy dialogue is an invaluable takeaway from the policy dialogue, as demonstrated in our study. Evidence has shown the usefulness of policy dialogues in promoting stakeholder involvement in translating evidence to policy [17, 18, 35, 36].

To improve future policy dialogues, the participants recommended retention of some features. In descending order of importance, these were the participatory and interactive engagement, involvement of all stakeholders, fair representation of stakeholders in the various subgroups during the deliberations, and well-itemized problems and policy recommendations. This differs from the findings of Lavis et al. [24], where the design features to be retained were, in descending order, skilled facilitation using an outside agent, bringing together all parties who could be affected by the outcome (as it allowed for a variety of perspectives and open dialogue), pre-circulation of packaged evidence summaries, alternative ways of addressing a policy issue, and the adoption of the Chatham House Rule. However, in a study by Boyko et al. [37], participants wanted all the design features in the deliberative dialogue retained in future dialogues.

With regard to what should change in future policy dialogues on the same issue, the most frequently demanded design feature change was the nonparticipation by the apex (political) policy-makers in the ministries such as the commissioners and permanent secretaries. Participants suggested that the participation of such political policy-makers should be made mandatory.

It is possible that this group of stakeholders could not attend the dialogue due to low prioritization of RI in the state and busy schedules. This is cause for concern considering the fact that the apex policy-makers are often too busy with other political engagements, making it difficult for them to sit in for such activities, and even when they attend they are distracted with many calls. One of the strategies used to ensure good attendance was early notification of the meeting followed by frequent calls and reminders. This highlights the need for continued advocacy to policy-makers to ensure they fully understand the importance of these KT processes.

As Boyko et al. [37] suggested, “fair representation among policy makers, managers, stakeholders and researchers” is a challenge for deliberative dialogues that address low-priority policy issues (i.e. those that are not on the “radar” of government decision-makers). For political officeholders, gains in RI may be considered as non-vote-catching given its nonphysical form compared to huge, more visible works like roads, bridges and buildings. This in turn could pose an implementation barrier to the policy at hand.

Participants’ written comments on important action they would personally do better or differently to address the featured policy issue reflects their different roles in the policy process but can be summarized to mean that they intend to use what they learned during the dialogue to improve RI service delivery. This is similar to the findings of other studies [37]. For example, some frontline health workers were willing to improve supportive supervision for the immunization programmes, civil society organizations suggested optimizing community resources and advocacy for RI, and the media committed to placing RI in the public domain. Thus the policy dialogue was an effective KT vehicle enabling policy actors to take responsibility to improve accountability in RI service delivery. This interesting finding highlights the value of personal responsibility in implementation of health programmes. Furthermore, the dialogue could potentially enhance evidence uptake, as has been demonstrated and advocated for by previous studies [25, 26, 38].

It is worth noting that these comments demonstrate the key roles of civil society organizations and the media in improving public policy, as has been described previously [39, 40].

Another interesting finding was that stakeholders wanted the brief circulated further in advance. This is surprising because, in our context, people do not tend to read materials sent to them in advance.

A major misgiving about implementing AFRIN in the state that was expressed by the health worker participants in this study was health worker-specific sanctions for poor RI performance without considering the failure of the government to budget for and/or release funds for RI services in a timely manner. They also wondered who would sanction the government when it defaults on its own responsibility. Research evidence has shown that non-budgeting and/or non-release of budgeted RI funds have been the most difficult bottleneck in RI service delivery in states and local government areas in Nigeria [2, 12, 13]. To mitigate this issue, the stakeholders suggested transforming the multi-stakeholder group into an RI “ombudsman”, in addition to prioritizing effective RI coverage as a political campaign issue in future elections.

## Conclusion

This study initiated the process of contextualizing to Ebonyi State the realities of the AFRIN national health policy document. It utilized a policy brief developed from a national document as a KT tool to convene a multi-stakeholder policy dialogue. Overall, the participants considered the policy brief to be of good quality and the dialogue useful for addressing a high-priority policy issue.

This study promoted an evidence-to-policy link by furnishing contextualized locally relevant evidence to policy-makers, who committed to incorporating them in the state’s RI accountability framework. To improve RI at the subnational level in Nigeria using the AFRIN framework, it is recommended that KT tools such as policy briefs and dialogue be deployed for multi-stakeholder engagement and advocacy.

Limitations of this study include limited generalizability to other contexts and the self-reported responses. However, this is one of the few studies in the African region assessing the use of KT tools and methods in engaging policy-makers towards operationalization of national policies at the subnational level.

## Data Availability

The datasets used and/or analysed during the current study are available from the corresponding author on reasonable request.

## Abbreviations

RI: Routine immunization
AFRIN: Accountability framework for routine immunization in Nigeria
UN: United Nations
